# FL3, a Synthetic Flavagline and Ligand of Prohibitins, Protects Cardiomyocytes via STAT3 from Doxorubicin Toxicity

**DOI:** 10.1371/journal.pone.0141826

**Published:** 2015-11-04

**Authors:** Rehana Qureshi, Onur Yildirim, Adeline Gasser, Christine Basmadjian, Qian Zhao, Jean-Philippe Wilmet, Laurent Désaubry, Canan G. Nebigil

**Affiliations:** 1 GPCRs in cardiobiology and Metabolism team, UMR 7242, CNRS–University of Strasbourg, LabEx Medalis, Strasbourg School of Biotechnology, Illkirch, France; 2 Laboratory of Therapeutic Innovation (UMR 7200), Faculty of Pharmacy, University of Strasbourg–CNRS, Illkirch, France; 3 Sino-French Joint Lab of Food Nutrition/Safety and Medicinal Chemistry, College of Biotechnology, Tianjin University of Science and Technology, Tianjin, 300457, China; National Institutes of Health, UNITED STATES

## Abstract

**Aims:**

The clinical use of doxorubicin for the treatment of cancer is limited by its cardiotoxicity. Flavaglines are natural products that have both potent anticancer and cardioprotective properties. A synthetic analog of flavaglines, FL3, efficiently protects mice from the cardiotoxicity of doxorubicin. The mechanism underlying this cardioprotective effect has yet to be elucidated.

**Methods and Results:**

Here, we show that FL3 binds to the scaffold proteins prohibitins (PHBs) and thus promotes their translocation to mitochondria in the H9c2 cardiomyocytes. FL3 induces heterodimerization of PHB1 with STAT3, thereby ensuring cardioprotection from doxorubicin toxicity. This interaction is associated with phosphorylation of STAT3. A JAK2 inhibitor, WP1066, suppresses both the phosphorylation of STAT3 and the protective effect of FL3 in cardiomyocytes. The involvement of PHBs in the FL3-mediated cardioprotection was confirmed by means of small interfering RNAs (siRNAs) targeting PHB1 and PHB2. The siRNA knockdown of PHBs inhibits both phosphorylation of STAT3 and the cardioprotective effect of FL3.

**Conclusion:**

Activation of mitochondrial STAT3/PHB1 complex by PHB ligands may be a new strategy against doxorubicin-induced cardiotoxicity and possibly other cardiac problems.

## Introduction

Anthracyclines (*e*.*g*., doxorubicin) remain a mainstay therapy for cancers such as leukemias, lymphomas, and breast and gastric cancers, even though these compounds cause substantial cardiotoxicity that can ultimately lead to congestive heart failure [1]. Therefore, approaches to alleviation of the cardiotoxic effects of doxorubicin are urgently needed in oncology. Dexrazoxane, which is the only clinically approved cardioprotectant against anthracycline cardiotoxicity, had been shown to induce secondary tumors and was consequently removed from the European market [2]. Thus, there is a need for efficacious and safe drugs that can protect cancer patients from the cardiotoxicity of anthracyclines.

Flavaglines are natural products isolated from Chinese medicinal plants that have potent anticancer effects without toxicity to healthy tissues [3]. Not only are flavaglines specifically toxic to cancer cells, but they also promote the survival of neurons, T lymphocytes, and cardiomyocytes under conditions of adverse effects of chemotherapeutic agents: cisplatin, etoposide, and doxorubicin respectively [4–6]. In particular, in a previous study, we found that a synthetic flavagline, FL3, almost doubles the survival rate of mice (56% treated versus 31% untreated) in an in vivo model of doxorubicin-induced acute cardiotoxicity [4]. Recently, we also showed that flavaglines directly bind to prohibitins (PHBs) in cancer cells [7]. PHBs are scaffold proteins that exist in two isoforms: PHB1 and PHB2 [8]. PHBs seem to perform a function in cancer cells that is different from that in healthy cells: PHBs may be located in several compartments, but they are mainly concentrated in mitochondria in healthy cells and in the nucleus in cancer cells [8]. This divergence of cellular localization (and possibly function) may explain why flavaglines promote apoptosis in cancer cells and survival in healthy cells.

PHB1 has been shown to prevent mitochondrial dysfunction via activating STAT3 in intestinal epithelium (as reviewed elsewhere [9]), but whether this event occurs in cardiomyocytes remains unreported. STAT3 phosphorylation [10] and overexpression [11] have been shown to protect the heart from doxorubicin-induced cardiotoxicity. Moreover, cardiac-restricted deletion of STAT3 increases the susceptibility to doxorubicin-induced heart failure [12, 13].

In this study our aim was to determine whether flavaglines exert their cardioprotective effect by modulating PHB1 localization and activating STA3 signaling.

## Methods

### Cell culture

The H9c2 cardioblast cell line that was derived from an embryonic rat heart was obtained from American Type Culture Collection (Manassas, VA, USA). The cells were grown in Dulbecco’s modified Eagle’s medium (DMEM) supplemented with 10% fetal calf serum at 37°C in a humidified atmosphere containing 5% CO_**2**_. The medium was changed every 2–3 days.

### The *in vitro* cardiotoxicity assay

H9c2 cells were plated and grown for 24 h in 100-mm culture dishes at 7 × 10^3^/cm^2^. Next, the cells were washed and cultured for 12 h in a glucose-free medium (Gibco; DMEM with L-glutamine, without D-glucose and sodium pyruvate) supplemented with only 1% fetal calf serum. The cells were pretreated with FL3 (100 nM) under serum-free conditions for 10 h, and then either doxorubicin (1 μM) or vehicle alone (DMSO) was added to the medium for additional incubation for 14 h. The doxorubicin concentration and incubation time were chosen in accordance with a known model of acute cardiotoxicity [14]. The H9c2 cardiomyocytes were preincubated with WB1066 (1 μM) for 1 h before FL3 treatment. The cells were then washed, and either terminal deoxynucleotidyl transferase dUTP nick end labeling (TUNEL) or fluorescence-activated cell sorting (FACS) analysis was performed.

### Detection and quantification of apoptosis

Terminal deoxynucleotidyl transferase dUTP nick end labeling (TUNEL) assays of fragmented DNA was performed according to according to the manufacturer’s instructions (Millipore) [4]. Cells were fixed in 4% formaldehyde, permeabilized. The cells were incubated with TdT terminal transferase and fluorescein-dUTP. Then, the cells were counterstained with 4’,6-diamidino-2-phenylindole (DAPI). The TUNEL labeling index was calculated as the percentage of DAPI-stained TUNEL-positive cells among total DAPI-labeled cells by viewing each visual field at 40× magnification. Generally, 10 different visual fields containing around 20 cells were analyzed in each sample, and each experiment was repeated at least three times.

Apoptosis was also analyzed by FACS analysis (FACSCalibur, Becton-Dickinson Biosciences, Le Pont De Claix, France). We harvested 7 × 10^3^ cells and washed them with “annexin binding buffer” (0.01 M HEPES, 0.14 M NaCl, 2.5 mM CaCl_2_) and labeled the cells with annexin V (dilution 1:50) and Topo (6.7 μg/mL). All assays were performed at least in triplicate, and the results were analyzed in the BD Cell Quest Pro software (Becton-Dickinson Biosciences).

### Pull-down assay

This assay was performed by means of FL3-Affigel as described previously [7]. One hundred million H9c2 cells were washed in PBS and lysed in 2 mL of a lysis buffer consisting of 50 mM Tris-HCl pH 8.0, 120 mM NaCl, 1% NP-40, 5 mM dithiothreitol (DTT), 200 μM Na_3_VO_4_, 25 mM NaF, and a protease inhibitor cocktail (Roche Diagnostics, Switzerland). Cellular debris were removed by centrifugation at 10 000 × *g* for 30 min. Five hundred micrograms of total protein extract was incubated for 12 h at 4°C with 40 μL of FL3-Affigel, negative control (NC)-coupled beads, or uncoupled Affi-Gel beads. The beads were extensively washed with the lysis buffer, and the bound proteins were eluted and reduced in a sample buffer consisting of 63 mM Tris-HCl pH 6.8, 2% SDS, 10% glycerol, a trace of bromophenol blue (0.05%), and 200 mM DTT for 30 min at 65°C. After cooling on ice, each sample was alkylated with a final concentration of 150 mM iodoacetamide for additional 30 min. The proteins were separated by SDS-PAGE (10% gel; Bio-Rad Laboratories, USA) and western blot analyses were performed using anti-PHB1 and anti-PHB2 antibodies.

### Immunohistochemical analysis

H9c2 cells were plated and grown for 24 h in Labtek-8 dishes at the density 2 × 10^4^/well in an incubator with 5% CO_2_ at 37°C. The medium was changed to DMEM containing 2% fetal calf serum for starvation of the cells for 24 h. After the starvation procedure, the cells were pretreated with MitoTracker Red CMXRos (Life Technologies) for 1 h, then treated with 0.1% dimethyl sulfoxide (DMSO) as a vehicle or FL3 (100 nM) for 0, 5, 10, 15, 30, 45, or 60 min. After that, the cells were fixed with 3.7% (v/v) formaldehyde for 15 min at room temperature and incubated with a blocking solution consisting of 5% BSA (bovine serum albumin) and 1% Triton X-100 in PBS at room temperature for 1 h. The cells were incubated with the anti-PHB1 antibody at 4°C overnight and then incubated for 1 h with an Alexa Fluor 488-conjugated anti-rabbit IgG antibody (Life Technologies/Molecular Probes) [15]. The cells were mounted on slides with the Vectashield Mounting Medium (Vector Labs) and DAPI for counterstaining of the nucleus. The cell images were acquired using a Leica TCS SP5 Confocal Microscopy System (Leica M, Germany) equipped with a 63×/1.40 NA oil-immersion objective lens. The images were captured at the scanning speed of 400 Hz and image resolution 512 × 512 pixels and were then analyzed using the Leica Application Suite, Advanced Fluorescence (LAS AF) software.

### Plasmid transfection

The PE935 (PHB1-Flag) and PE936 (PHB2-Flag) plasmids were transfected into H9c2 cells using Jet Prime (POL114-07, PolyPlus Transfection). The cells at 60–80% confluence in a 60-cm^2^ culture plate were incubated with an antibiotic-free medium. Twelve micrograms of plasmid DNA was used for the transfection. Forty-eight hours after the transfection, the FLAG-PHB1 and FLAG-PHB2 proteins were purified from the transfectant H9c2 cells by immunoprecipitation using an anti-FLAG antibody and FLAG-peptide elution.

### Protein purification by immunoprecipitation

Immunoprecipitation of PHBs with the anti-FLAG antibody from the transfectant H9c2 cells was performed as described previously [7]. The H9c2 cells were incubated for 0, 15, or 30 min with FL3 (Enzo Life Sciences). Subsequently, the cells were washed in ice-cold PBS, lysed in the IP buffer (20 mM Tris-HCl, 5 M NaCl, 2 mM EDTA, 1% Triton X-100, and protease inhibitors) and centrifuged (10 000 × *g*, 20 min) to clear the lysates. Aliquots were taken for input control, and the lysates were incubated with protein G Plus/A-agarose beads (#IP10, Calbiochem) for 30 min at 4°C, then overnight with an anti-FLAG antibody (anti-FLAG M2, Sigma-Aldrich, St. Louis, MO, USA; cat. # F1804). After that, the immunoprecipitates were washed with a lysis buffer (1% NP-40, 300 mM NaCl, 10% glycerin, 10 mM Tris-HCl pH 7.5), then a buffer without salt (1% NP-40, 10% glycerin, 10 mM Tris pH 7.5), and centrifuged for 10 min at 20 000 rpm and 4°C. Next, the samples were boiled in a denaturing sample buffer at 95°C for 5 min. The binding of STAT3 to the PHB1 proteins was detected by western blot analyses using anti-STAT3 or anti-phospho-STAT3 antibodies (Cell Signaling).

### Subcellular fraction of H9c2 cells and the STAT3 phosphorylation assay via western blotting

H9c2 cells were plated and grown for 24 h. Next, the cells were washed and cultured for 12 h in the above-mentioned glucose-free medium, supplemented with only 1% fetal calf serum. The cells were then incubated with either FL3 or vehicle alone (0.1% DMSO) for 0, 5, 10, 15, or30 min and harvested with a lysis buffer (50 mM Tris-HCl pH 7.0, 1 mM EDTA, 100mM NaCl, 0.1% SDS, 1% NP-40, 1 mM Na_3_VO_4_, 1 mg/mL aprotinin, 1 mg/mL pepstatin, and 1 mg/mL leupeptin). The whole-cell lysates were centrifuged at 12 000 × g for 15 min at 4°C. The cell debris was removed.

Cytoplasmic and mitochondrial fractions from cultured cells were prepared using Subcellular Protein Fractionation Kit for Cultured Cells (Thermo Scientific) and nuclear isolation kit, employing the nuclear protein extraction buffer (20 mM Tris–HCl, pH 7.6, 50 mM KCl, 400 mM NaCl, 1 mM EDTA, 0.2 mM PMSF, 5 mM β-mercaptoethanol, aprotinin (1000 U/ml), 1% Triton X-100, and 20% glycerol as described [16]. 30μg of total protein, 5μg or 10μg of cytosolic, mitochondrial proteins or nuclear protein were used for Western blot analyses. The proteins were separated under denaturing conditions using SDS-PAGE (10% gel) and transferred to a polyvinylidene difluoride (PVDF) membrane. The blots were incubated with a blocking solution consisting of a 5% solution of a fat-free milk powder in PBS-T (PBS plus Tween 20, 0.1%) at room temperature for 1 h. After three washes with PBS-T for 10 min, the blots were incubated overnight at 4°C with gentle shaking with a primary antibody anti-phospho-STAT3 antibody Ser (727) (Cell Signaling), (1:500 dilution in PBS-T containing 0.5% of the fat-free milk powder) or PHB1 (Cell Signaling).

After three washes with PBS-T, the membrane was incubated for 1 h at room temperature with gentle shaking with a horseradish peroxidase-conjugated goat anti-IgG antibody (1:1000 dilution) in PBS-T containing 0.5% of the fat-free milk powder. The expected bands were visualized after 5-min incubation to induce enzyme-linked chemiluminescence (GE HealthCare), and then the blots were washed, stripped, and reprobed with a Total -STAT3 antibody (Cell Signaling) or vinculin (Cell Signaling) or actin (Santa Cruz) as internal control, followed by incubation with a suitable secondary antibody. The phospho-STAT3 or PHB1 signals were quantified by scanning laser densitometry and normalize to total amounts of the corresponding STAT3 or vinculine protein, respectively.

### Transfection with small interfering RNA (siRNA)

A 50-nM solution of siRNA against rat PHB2 (Ambion; siRNA #258474) or a mixture (10 nM each) of siRNAs against rat PHB2 and PHB1 (Ambion, USA; siRNA #199561) were used for transfection of 90%- to 95%-confluent cells in a serum-free medium. Nonspecific siRNA (Ambion) served as a negative control. The transfection was based on Lipofectamine 2000 (Invitrogen, USA), according to the manufacturer’s instructions. Forty-eight hours after the transfection, the PHB1 levels were measured by quantitative PCR and western blot analysis.

### Statistical analysis

All samples were prepared (and used in experiments) at least in triplicate. The results of the quantitative experiments were expressed as mean ± SEM. Multigroup comparisons were performed using one-way analysis of variance (ANOVA) with *post hoc* Bonferroni’s correction. Comparisons between two groups were conducted using unpaired Student’s *t* test. In all analyses, p < 0.05 was assumed to denote statistical significance. All calculations were performed in the Prism software.

## Results

### FL3 binds to PHB1 and PHB2 in cardiomyocytes

**Fig 1:**
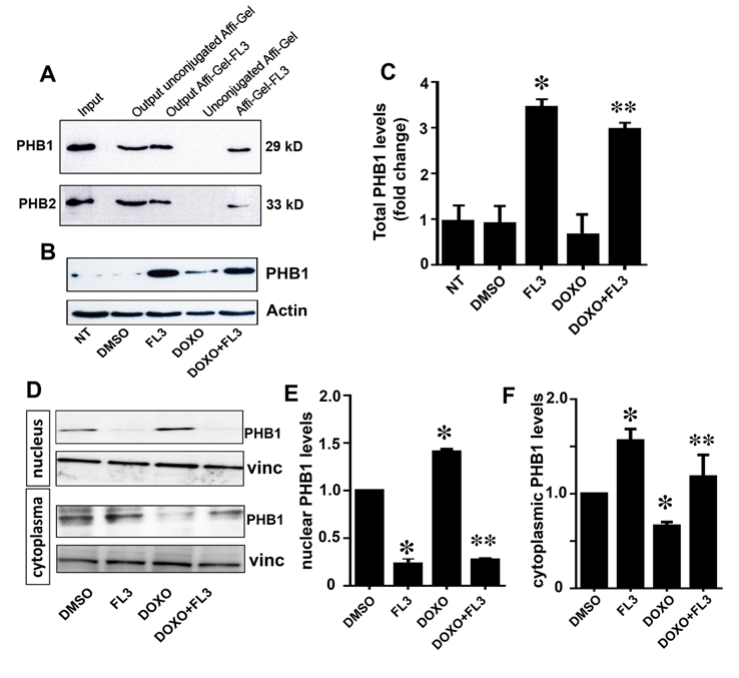
Synthetic flavagline (FL3) binds PHB1 and PHB2 and increases PHB1 leves in H9c2 cells. **A.** Whole-cell extracts of the H9c2 line (input) were either incubated with the beads Affi-Gel 10 conjugated with FL3 or blocked with ethanolamine (unconjugated Affi-Gel) [7,17]. The bound and eluted proteins (Affi-Gel-FL3) and output proteins (output Affi-Gel-FL3) were analyzed by western blotting using antibodies against PHB1 and PHB2 (*n* = 3). **B and C.** Representative western blot analyses and histogram based quantification of total PHB1 levels in the cell lysates by. FL3 alone increased PHB1 protein levels within 10h as compare to non-treated cells (NT). However PHB1 level was lower in the present of both FL3 and doxorubicin (n = 3). **D and E.** Representative western blot analyses and histogram based quantification of nuclear PHB1 levels by. Doxorubicin accumulates PHB1 in the nucleus but lowers in the cytoplasm that was reduced by preconditioning with FL3. * Indicates p<0.05 as compare to control, ** indicates p<0.05 as compare to the doxorubicin alone group.

To test whether FL3 binds to PHBs in cardiomyocytes, we performed a pull-down assay with protein extracts of the H9c2 cardiomyocytes using a biologically active flavagline (FL3) conjugated to Affi-Gel beads [7,17]. Whole-cell extracts from H9c2 cells (input), the bound and eluted proteins (Affi-Gel-FL3), and output proteins (output Affi-Gel-FL3) were subjected to western blot analysis using antibodies against PHB1 and PHB2. Both PHB1 and PHB2 (Fig 1A) were retained by the affinity matrix. The blank beads did not pull down any PHB proteins (lane 4 in Fig 1A). These data showed that PHB1 and PHB2 were the cellular targets of FL3 in the H9c2 cardiomyocytes. Next, we examined whether doxorubicin and/or FL3 modify the content of PHB1 in these cells (Fig 1B and 1C). FL3 treatment of the H9c2 cells for 10h (with or without doxorubicin) greatly augmented PHB1 protein levels. Doxorubicin has no significant effect on PHB1 levels (Fig 1B and 1C), but it induced its accumulation in the nucleus (Fig 1D, 1E and 1F). This translocation of PHB1 from cytoplasm to the nucleus was blocked by FL3 (Fig 1D, 1E and 1F). This data indicate that PHB1 levels and localization can be greatly modified by FL3 treatment.

### FL3 promotes the localization of PHB1 to mitochondria

**Fig 2:**
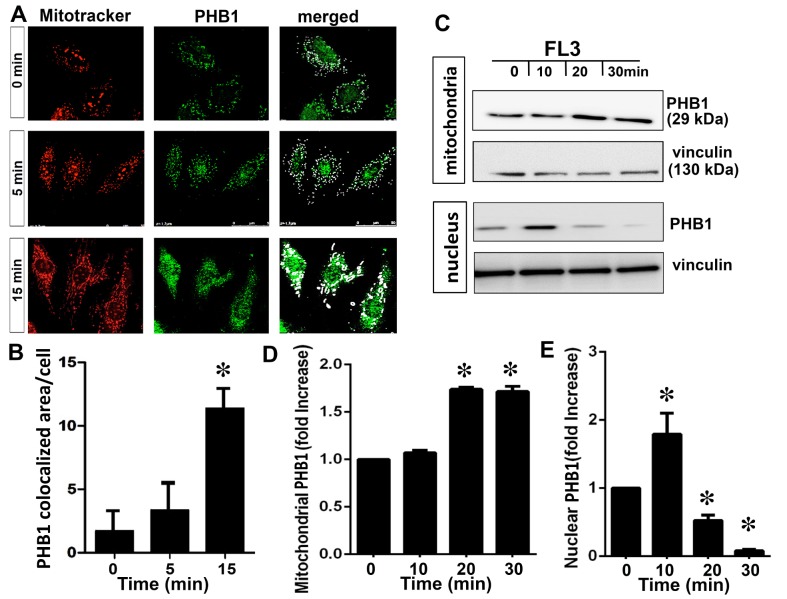
The flavagline FL3 induces translocation of PHB1 to mitochondria in cardiomyocytes. **A.** H9c2 cells were incubated with FL3 (100 nM) and analyzed by confocal microscopy. The cells were co-labeled with the anti-PHB1 antibody (green staining), mitotracker (red staining), and (DAPI; blue staining). The latter two dyes stained mitochondria and the nucleus, respectively. Merged confocal images show that FL3 induced the translocation of PHB1 to mitochondria (white arrows show PHB1 and mitotracker co-localization). **B.** The histogram shows quantitative analyses of co-localization of PHB1 and Mito Tracker in each cell by confocal analyses (n = 6). **C.** Representative illustration of PHB1 levels in mitochondrial and nuclear fractions upon FL3 treatment. In the mitochondrial fraction PHB1 accumulation by FL3 occurred within 20 min. PHB1 was initially increased in nucleus and rapidly reduced within 20 min. **D and E.** The histogram shows quantitative analyses of mitochondrial and nuclear PHB1 levels upon treatment of H9c2 cells with FL3 (100 nM). * Indicates p<0.05 as compare to vehicle (n = 3).

A large body of evidence suggests that the subcellular location of PHBs determines whether a PHB protein promotes apoptosis or cytoprotection [8,18]. Accordingly, we examined the intracellular localization of PHB1 after treatment with FL3 to gain some insight into FL3’s mechanism of action. H9c2 cells were double-labeled with an anti-PHB1 antibody and the mitochondrial dye Mitotracker Red. PHB1 was detected throughout the cytoplasm of the H9c2 cardiomyocytes in the basal condition (Fig 2A). The staining pattern for PHB1 maximally matched that of Mitotracker Red after 15-min incubation of the cells with FL3, indicating that FL3 promoted PHB1 accumulation predominantly in mitochondria (Fig 2B). Mitochondrial fraction of the FL3 treated H9c2 cells confirmed an amplification of PHB1 levels in mitochondria after 15 min (Fig 2C and 2D). PHB1 accumulation in nucleus was elevated within 10 min and was consequently reduced within 20 min upon FL3 treatment (Fig 2C and 2E). This data clearly showed that FL3 promotes nuclear translocation of PHB1 to mitochondria.

### FL3 promotes activation of STAT3 by PHB1

**Fig 3:**
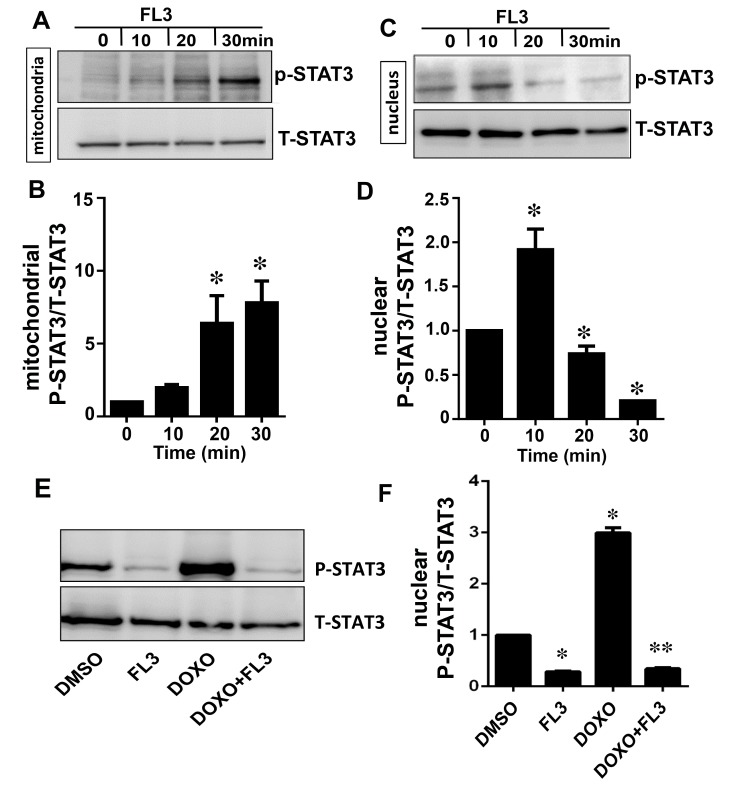
The mitochondrial STAT-3 phosphorylation is correlated with PHB1 translocation to mitochondria by FL3 in cardiomyocytes. **A and B.** Representative western blot analyses and histogram based quantification of mitochondrial STAT3 activation by phosphorylation. STAT3 was phosphorylated by FL3 in mitochondrial fraction. **C and D.** Representative western blot analyses and **h**istogram based quantification of nuclear STAT3 activation by phosphorylation. STAT3 activation was only detected in nucleus after FL3 treatment. **E and F.** The western blot and histogram show quantitative analyses of nuclear phosphorylated STAT3 levels upon treatment of H9c2 cells with control (DMSO), FL3 (100 nM), doxorubicin (1μM), and FL3 + doxorubicin. Doxorubicin elevated phosphorylated STAT3 levels, which were reduced by FL3 (*n* = 3; *p < 0.05, compared to vehicle; **p < 0.05, compared to doxorubicin treatment).

FL3 enhanced phosphorylation of STAT3 in mitochondria in time dependent manner (Fig 3A and 3B) and correlated with PHB1 accumulation in the mitochondria and nucleus (Fig 3C and 3D), indicating that FL3 promotes nuclear translocation of PHB1 to mitochondria and consequently STAT3 phosphorylation. Next we investigated whether PHB1 accumulation and STAT3 phosphorylation are also correlated upon doxorubicin treatment of the H9c2 cells. Accordingly, phosphorylation of nuclear STAT3 is elevated by doxorubicin that was blocked by FL3 preconditioning (Fig 3E and 3F).

**Fig 4:**
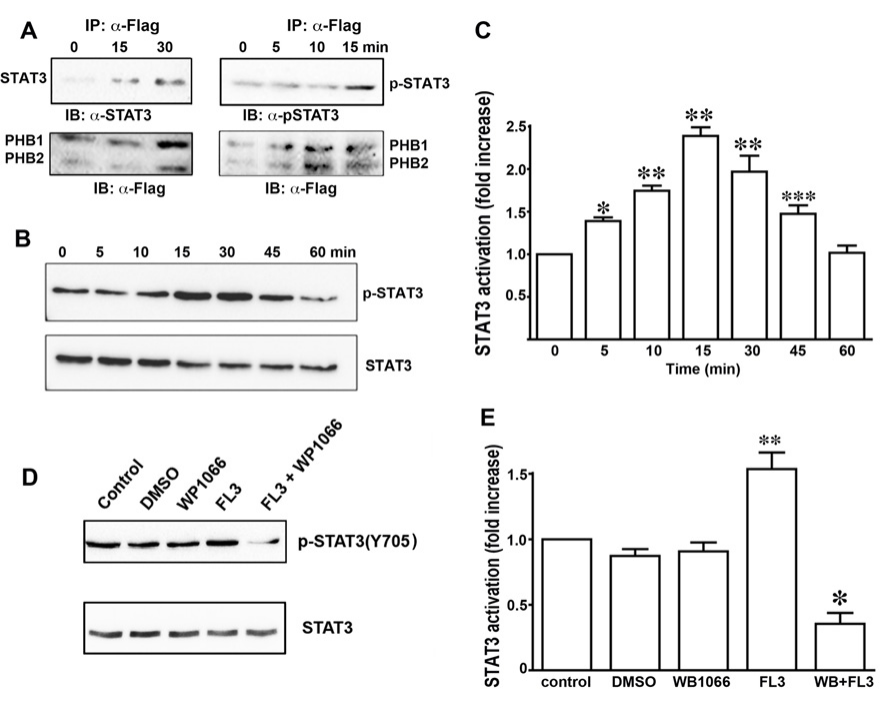
FL3 rapidly induces phosphorylation of STAT3. **A.** STAT3 coimmunoprecipitates (co-IP) with PHB1. An anti-FLAG antibody was incubated with extracts of the H9c2 cardiomyocytes. Immunoprecipitates were resolved by means of SDS-PAGE and probed for STAT3 and the FLAG tag to detect both PHB1 and PHB2. **B.** Representative western blots of protein lysates of H9c2 cells treated with FL3 (100 nM), by means of antibodies that recognize either phosphorylated (Tyr^705^) or total STAT3 protein. **C.** Quantitative analysis of the western blots (percentage of phosphorylated STAT3 in total STAT3, *n* = 4; *p < 0.05, compared to control; **p < 0.001, compared to control; ***p < 0.01, compared to control). **D** and **E.** Effects of the Janus kinase 2 (JAK2) inhibitor WP1066 on STAT3 phosphorylation: Representative western blots and quantitative analysis (percentage of phosphorylated STAT3 in total STAT3, *n* = 4; p < 0.05, compared to control; **p < 0.05, compared to FL3).

Next, we addressed whether FL3 promotes interaction of PHB1 with STAT3 in H9c2 cells, since PHB1 accumulation and STAT3 phosphorylation are correlated in mitochondria and nucleus. Moreover, PHB1 has been shown to heterodimerize with STAT3 [8]. H9c2 cells that were cotransfected with plasmids encoding FLAG-tagged PHB1 and PHB2 were incubated with FL3 (100 nM) for immunoprecipitation of PHBs with an anti-FLAG antibody. The immunoprecipitated cell lysates (input) from the H9c2 cells that were transfected with PHB1 or PHB2 (IP-α-Flag) were subjected to western blot analyses with anti-flag antibodies. Significant coimmunoprecipitation of PHB1 with STAT3 was observed at the data point 15 min after FL3 treatment (Fig 4A, left panels), whereas interactions of PHBs with either AKT or ERK were not detected. These results showed that FL3 induced heterodimerization of STAT3 with PHBs. When the immunoprecipitated PHB1 proteins were visualized with an antibody recognizing the phosphorylated form of STAT3, the phospho-STAT3 was significantly upregulated 15 min after FL3 treatment (Fig 4A, right panels). Taken together, these data suggested that PHBs interacted with the STAT3 protein, and this interaction induced activation of STAT3 by phosphorylation.

Next, we examined STAT3 activation by phosphorylation in H9c2 cells after FL3 treatment. Using a specific anti-phospho-STAT3 antibody and western blot analysis, we found that FL3 (100 nM) rapidly promoted the phosphorylation of STAT3 in the H9c2 cardiomyocytes: the phosphorylation reached a maximum within 15 min (Figs 4B and 3C). To gain further insight into the activation of STAT3 by PHBs, we tested whether the STAT3 phosphorylation is inhibited by WP1066 [19], an inhibitor of the JAK2 kinase (this reagent is commonly used to block STAT3 activation). WP1066 at 100 nM significantly inhibited the FL3-induced STAT3 activation (Figs 4D and 3E).

### FL3 triggers cardioprotective signaling by targeting PHB1 and its signaling

**Fig 5:**
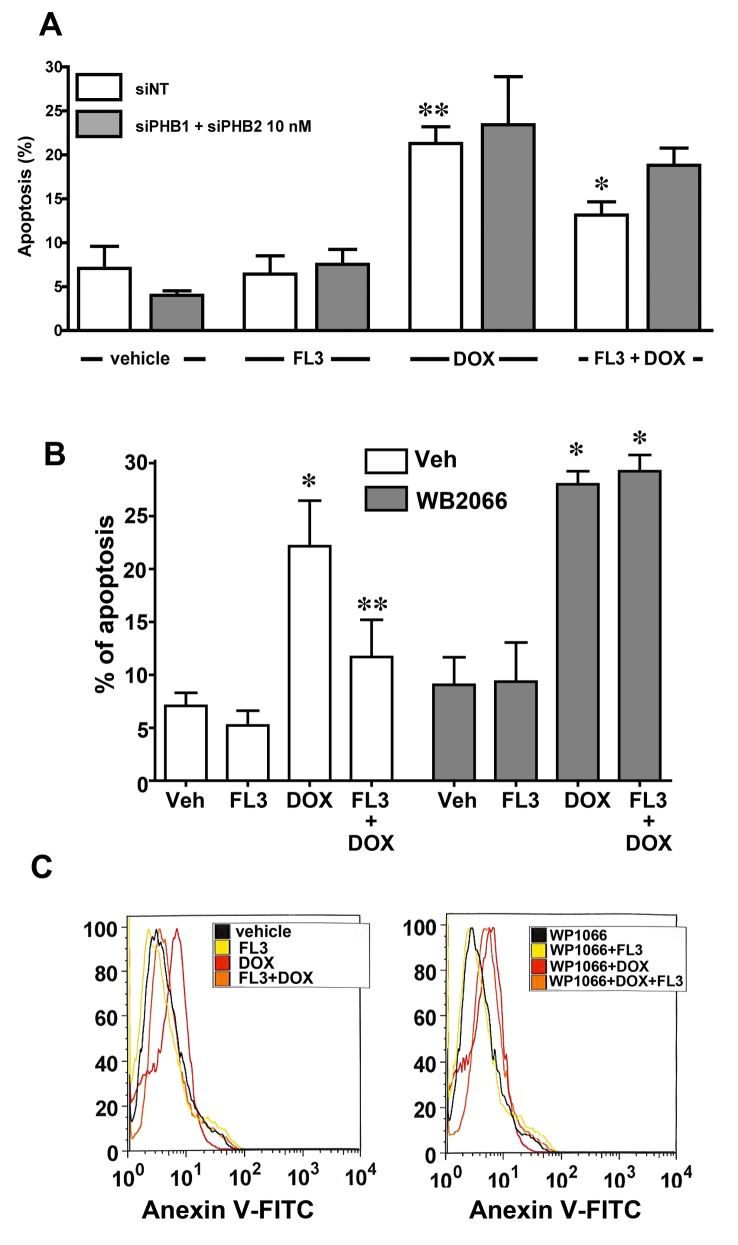
FL3 protects H9c2 cardiomyocytes by acting on PHBs and their signaling target STAT3. **A.** The histogram shows the percentage of apoptotic cells induced by doxorubicin (1 μM) among H9c2 control cells (transfected with nonspecific small interfering RNA [si-NT]) or the H9c2 cells where PHB1 or PHB2 were downregulated using specific small interfering RNA (siRNA). Knocking PHB1 or PHB2 down greatly diminished the cardioprotective effect of FL3 (100 nM; *n* = 4 to 5; *p < 0.05, compared to vehicle; **p < 0.05, compared to doxorubicin (doxo). **B.** The TUNEL assay shows the percentage of apoptotic cells in the 4’,6-diamidino-2-phenylindole (DAPI)-positive total cell population. **C.** Fluorescence-activated cell sorting (FACS) analysis shows the percentage of the maximum among annexin V-positive cells (*n* = 3; *p < 0.05, compared to vehicle; **p < 0.05, compared to doxorubicin treatment).

To confirm the involvement of PHBs in the mechanism of action of FL3, we tested whether a knockdown of PHB1 in cardiomyocytes affects the cardioprotective effect of FL3. Accordingly, we transfected the H9c2 cardiomyocytes with siRNAs against PHBs to downregulate PHBs without altering cell survival. Of the two anti-PHB siRNA sequences tested, si-PHB1 at 50 nM or si-PHB1 together with si-PHB2 (10 nM each) downregulated both PHB1 mRNA and protein: by 80% and 70%, respectively. The siRNA-mediated downregulation of PHB1 and PHB2 significantly reduced the cytoprotective effect of FL3 (Fig 5A), whereas transfection with control (nonspecific) siRNA did not. These data indicated that PHB1 and PHB2 were both involved in the mechanism of action of FL3.

To determine whether the STAT3 phosphorylation in cardiomyocytes was indeed involved the cardioprotective mechanism; we compared the apoptosis levels in TUNEL and FACS assays when the H9c2 cardiomyocytes were pretreated with WP1066 or vehicle alone. The TUNEL (Fig 5B) and FACS data (Fig 5C) revealed that WP1066 strongly attenuated the cardioprotective effect of FL3. Overall, these results supported the notion that phosphorylation of STAT3 is a crucial step in the mechanism of cardioprotective action of FL3.

### The PHB1/STAT3 complex is a key participant in the FL3-activated STAT3 pathway

**Fig 6:**
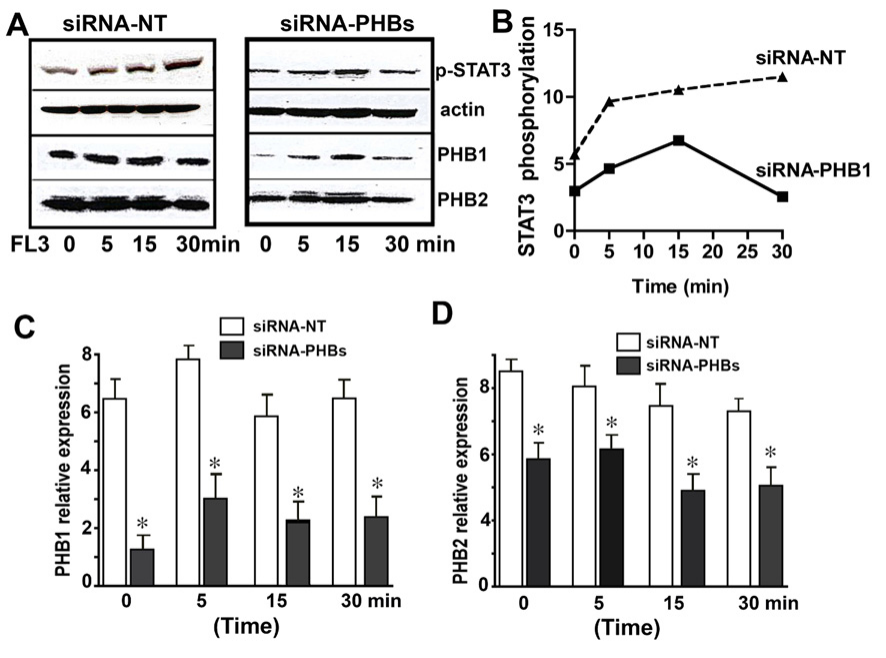
Small-interfering-RNA (siRNA)-mediated downregulation of PHB1 proteins attenuates FL3-induced cardioprotection from doxorubicin toxicity. **A.** Representative western blots show induction of STAT3 phosphorylation by the synthetic flavagline (FL3) in H9c2 cells transfected with nonspecific siRNA (left) or with anti-PHB1 siRNA (right). **B.** Quantification of phosphorylated STAT3, with normalization to actin (*n* = 3; *p < 0.05, compared to vehicle). FL3-mediated STAT3 activation by phosphorylation was abolished when the expression of PHBs were reduced. **C.** This histogram shows downregulation of PHB1 after transfection of H9c2 cells with anti-PHBs siRNA (*n* = 3; *p < 0.05, compared to vehicle). **D.** This histogram shows downregulation of PHB2 after transfection of H9c2 cells with anti-PHBs siRNA (*n* = 3; *p < 0.05, compared to vehicle).

To confirm whether the PHB1/STAT3 complex is involved in the FL3-activated STAT3 pathway, we transiently cotransfected the cells with either anti-PHB1 siRNAs or scrambled RNA as a control, then incubated the cells with FL3, and tested them for STAT3 phosphorylation. The anti-PHB1 siRNA, but not scrambled RNA, strongly attenuated the FL3-induced STAT3 phosphorylation (Fig 6A and 6B). The levels of PHBs in siRNA-nontargeted and siRNA-PHBs transfected cells are shown in (Fig 6C and 6D). These results showed that PHB1 was necessary for the STAT3 activation by FL3, and that STAT3 was downstream of PHB1 in the FL3-mediated survival pathway.

## Discussion

**Fig 7:**
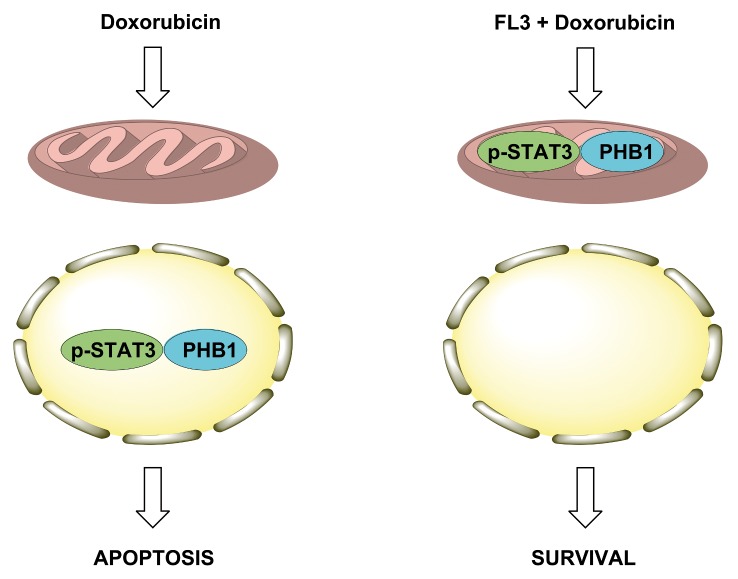
Proposed mechanism of FL3-induced cardioprotection from doxorubicin toxicity. Doxorubicin induces the translocation of PHB1 and phosphorylated STAT3 in the nucleus of cardiomyocytes to induce apoptosis. On the opposite, FL3 induces the translocation of these signaling proteins into mitochondria to protect the cell against the adverse effects of doxorubicin.

A study on the cardiac phosphoproteome has already shown PHB1 to be a prime target of doxorubicin [20]. Nonetheless, how the PHB proteins participate in the survival mechanisms against doxorubicin-mediated cardiotoxicity was not known. Here, we show for the first time that FL3 binds to PHBs and translocated PHB1 to mitochondria. Accumulation of PHB1 in mitochondria is associated with STAT3 phosphorylation. It seems that mitochondrial PHB1 accumulation stabilizes mitochondrial membrane, activates mitochondrial STAT3 activation and initiates FL3-mediated cardioprotection. On the opposite, doxorubicin provokes the PHB1 accumulation and STAT3 phosphorylation in nucleus, leading to cardiomyocyte apoptosis (Fig 7).

PHB1 has been reported to promote the survival of many noncancerous cell types, including cardiomyocytes [8,21–26]. Overexpression of PHB1 inhibits the mitochondria-mediated apoptosis pathway in H9c2 cells that is induced by hypoxia. Reduced levels of transcripts and mitochondrial PHB1 proteins were found in the left ventricle of spontaneously hypertensive rats. Heart-specific PHB1-transgenic mice show low levels of apoptosis and mitochondrial fission in the heart, and consequently, a smaller myocardial infarction size after an experimental infarction [20]. Proteomics studies have shown that PHB1 expression increases dramatically in cardiomyocytes mitochondria after chronic restraint stress [27]. H_2_O_2_-induced oxidative stress increases also the mitochondrial content of PHB1 in cardiomyocytes to stabilize mitochondrial membrane potential, inhibit the release of cytochrome c from mitochondria and maintain the mitochondrial function assessed by the preservation of the H^+^-ATPase activity [28]. These data indicate that, in mitochondria, PHB1 is a critical factor that protects cardiomyocytes from oxidative stress.

We found here that in cardiomyocytes, FL3 promotes translocation of PHB1 to mitochondria. This observation is in line with other studies showing that translocation of PHB1 from the nucleus to mitochondria is necessary for cytoprotection in ovarian granulosa cells [23,24], pancreatic β-cells [25], and the retinal epithelium [26]. Accumulation of PHB1 in the mitochondrial membrane can stabilize this membrane, blocking the apoptotic machinery.

Several studies indicate that during apoptosis induced by cytotoxic agents PHB1 migrates to the nucleus where it co-localizes with p53 [29, 30]. Interestingly, we found that doxorubicin does not alter total PHB1 levels in H9c2 cells, but promotes accumulation of PHB1 in the nucleus. This effect was abolished by FL3 treatment, which induced the translocation of PHB1 to mitochondria. The total PHB1 levels in H9c2 cells were also significantly induced by FL3 treatment (for 10h) that was reduced by doxorubicin treatment. The increase of PHB1 expression levels could be due to the activation of STAT3 during the preconditioning by FL3. Indeed, STAT3 is known to upregulate PHB1 during oxidative stress [31].

Theiss and collaborators demonstrated that PHB1 induces phosphorylation of STAT3, thereby stimulating its interaction with PHB1 in mitochondria and ensuring consequent protection of intestinal epithelial cells from TNF-α-induced mitochondrial stress and apoptosis [9]. Such a cytoprotective mechanism has not been reported yet in any other cell types. Consistent with the above observations, our results show that in cardiomyocytes, FL3 induces rapid translocation of PHB1 to mitochondria simultaneously with STAT3 phosphorylation.

STAT3 is a transcription factor that drives expression of antiapoptotic and antioxidant genes [32,33]. STAT3 promotes cardiomyocytes survival through 2 types of actions: -in the nucleus it acts as transcription factor to upregulates iNOS and COX-2 and stimulates the adaptation of the heart to ischemic stress [34]. In mitochondria, STAT3 prevents mitochondria-mediated apoptosis, inhibits the opening of mitochondrial permeability transition pores (MPTP) [32] and modulates the electron transport chain [35]. STAT3 phosphorylation [10] and overexpression [11] have been shown to protect cardiomyocytes from apoptosis induced by doxorubicin in heart tissues.

We demonstrated that inhibition of STAT3 activation by WP1066 blocks the cardioprotective effect of FL3, thus confirming that STAT3 activation is essential for prevention of cardiomyocyte death. The mechanistic link between the activation of STAT3 and PHB1 is currently not clear. Both proteins form a complex in cardiomyocytes 15 min after initiation of FL3 treatment—when STAT3 is maximally phosphorylated—suggesting that both events are connected. It is therefore tempting to hypothesize that STAT3 becomes phosphorylated when it interacts with PHB1, especially because FL3 cannot induce STAT3 phosphorylation or protect cardiomyocytes from doxorubicin toxicity in PHB1-deficient cells.

This study seems to provide the first evidence that targeting of PHB1 by small molecules such as FL3 induces cytoprotection via activation of STAT3 signaling in mitochondria. This strategy may turn out to be a valid therapeutic method for protection of the myocardium from anthracycline-induced cardiotoxicity and ischemia/reperfusion-mediated damage. The beneficial effects of mitochondrial STAT3 in the heart have now been demonstrated, but the currently used methods for activation of mitochondrial STAT3 signaling are not very convenient in terms of clinical application, because of lack of specific activator. Indeed, G-CSF, EPO, and IL-11 protect the heart from ischemic injury, doxorubicin cardiotoxicity, or cardiac fibrosis utilizing mitochondrial STAT3 signaling pathway, however they also activate other signaling pathways that may induce adverse effects [36–38]. Nevertheless, as far as we know, small molecules, such as FL3, have not been reported to activate STAT3 in the heart.

In summary, mitochondrial versus nuclear PHB/STAT3 complex is critical for the cardioprotective effect of FL3 (Fig 7). Because of the importance of STAT3/PHB1 complex in mitochondria as a therapeutic target in heart failure, the effects of flavaglines need to be examined in experimental models of this disease.
